# Computer Modeling of Clonal Dominance: Memory-Anti-Naïve and Its Curbing by Attrition

**DOI:** 10.3389/fimmu.2019.01513

**Published:** 2019-07-03

**Authors:** Filippo Castiglione, Dario Ghersi, Franco Celada

**Affiliations:** ^1^Institute for Applied Computing, National Research Council of Italy, Rome, Italy; ^2^School of Interdisciplinary Informatics, College of Information Science and Technology, University of Nebraska at Omaha, Omaha, NE, United States; ^3^NYU School of Medicine, New York, NY, United States

**Keywords:** computer modeling, IMMSIM, memory-anti-naïve, attrition, CD8+ response

## Abstract

Experimental and computational studies have revealed that T-cell cross-reactivity is a widespread phenomenon that can either be advantageous or detrimental to the host. In particular, detrimental effects can occur whenever the clonal dominance of memory cells is not justified by their infection-clearing capacity. Using an agent-based model of the immune system, we recently predicted the “memory anti-naïve” phenomenon, which occurs when the secondary challenge is similar but not identical to the primary stimulation. In this case, the pre-existing memory cells formed during the primary infection may be rapidly deployed in spite of their low affinity and can actually prevent a potentially higher affinity naïve response from emerging, resulting in impaired viral clearance. This finding allowed us to propose a mechanistic explanation for the concept of “antigenic sin” originally described in the context of the humoral response. However, the fact that antigenic sin is a relatively rare occurrence suggests the existence of evolutionary mechanisms that can mitigate the effect of the memory anti-naïve phenomenon. In this study we use computer modeling to further elucidate clonal dominance and the memory anti-naïve phenomenon, and to investigate a possible mitigating factor called attrition. Attrition has been described in the experimental and computational literature as a combination of competition for space and apoptosis of lymphocytes via type-I interferon in the early stages of a viral infection. This study systematically explores the relationship between clonal dominance and the mechanism of attrition. Our results suggest that attrition can indeed mitigate the memory anti-naïve effect by enabling the emergence of a diverse, higher affinity naïve response against the secondary challenge. In conclusion, modeling attrition allows us to shed light on the nature of clonal interaction and dominance.

## Introduction

Immunological memory, which appeared in the adaptive immune system roughly 600 million years ago, resulted in a substantial evolutionary advantage for vertebrates, whose immune systems acquired the ability to “remember” infectious agents and rapidly deploy effector cells in subsequent encounters with the same microorganisms or viruses. However, the tendency of many infectious agents to mutate can reduce the efficacy of memory cells, whose affinity for mutated antigens can drastically decrease. Therefore, between the two extremes of the *homologous* challenge (with identical primary and secondary infections) and independent primary responses against two unrelated infectious agents, there exists a wide range of responses where the host has partial immunity against the new infection. Interestingly, partial immunity can exist not only between different strains of the same microorganism or virus, but also between apparently unrelated viruses, as shown by the pioneering work of Selin and Welsh in the field called *heterologous immunity* (1).

Cross-reactive immune responses against different viruses are believed to be ubiquitous, and can have beneficial, neutral, or detrimental effects for the host, in ways that are not easy to predict. Detrimental effects of partial immunity were described by Fazekas de St. Groth in 1966, when highest lethality rates were found among patients with a history of past encounters with far cross-reactive infectious agents. This phenomenon was studied in the humoral branch of the adaptive immune system and was labeled “original antigenic sin” (2). When cross-reactivity is too weak to cure the infection, the thwarting of naïve responses by memory is still blocking the development of the primary response, adding failure to failure: failure to cure and failing blocking the default defense.

The patterns of viral mutations and cross-reactive interactions are difficult to trace and define *in vivo*, making computational modeling highly beneficial. In a previous study we systematically studied the effect of a stepwise increase of the distance between two antigens subsequently injected in an *in silico* model (3). Unexpectedly, we identified an intermediate range of priming-challenge antigenic distances where memory is unable to mount an efficient defense, but it still outcompetes the primary response. Further *in silico* experimentation corroborated our first studies proving that the mechanism of memory anti-naïve (MaN) is fueled by the specific competition for antigen (4). Competition for antigen plays a key role in allowing high affinity clones to emerge in an immune response. However, memory has a faster dynamic than a primary response. In the early phases of an infection—while the primary response is still not ready to engage—the quantity of available antigen is growing but still limited. Thus, low affinity memory cells can potentially outcompete naïve cells, resulting in an immune response of lower quality.

Recently, Welsh et al. described a mitigating phenomenon named *attrition*, which is triggered by competition for space among clones of immunocytes at the time of antigen contact in a lymph node (5). Attrition is driven by short-distance effect of IFN-β that induces apoptosis on cytotoxic T-cells (Tc) by contact. The net effect is to reduce the growing lymphoid Tc population, and thus to favor the fittest cell lines in terms of affinity against the invader. The present *in silico* study is focused on modeling the mechanism of attrition and measuring its effects on the speed and on the affinity of the secondary response while systematically varying the degree of cross-reactivity.

## Background

### Nature and Role of Computational Models

The biology of the immune response has been studied intensively in the few decades before and after the turn of the century and we witnessed an extraordinary growth in the number of researchers worldwide. As a result, we witnessed an exponential increase in the data being generated, resulting in the need for computational models to help make sense of it.

Computational modeling of the immune system experienced a strong burst in the 1980s, when several interdisciplinary collaborations brought together immunologists and mathematicians of various shades. These collaborations were fostered by two breakthrough events in the theoretical immunology community that had been engaged in adaptive immunology for some decades: the first solved the genetic problem of immune diversity (6); the second explained the formation of synapses between lymphocytes, allowing cell cooperation in most actions of the immune system (7). These achievements increased the size and the complexity of the field. At the same time, they created space for computational modeling.

Agent-based modeling is a relatively novel paradigm of modeling that satisfies the requirements of simplicity and parsimony in the description of a phenomenon by emphasizing first principles. It is a general modeling paradigm for complex systems inspired by von Neumann's “cellular automata” (8). Agent-based models consist of discrete dimensional space and time scales, where agents are, in our case, the relevant cells (or molecules) equipped with virtual receptors and capabilities, which reflect experimental observations.

The computational model C-IMMSIM, as well as the pioneering IMMSIM (9, 10), has been conceived to allow the dynamic representation of hypotheses and their preliminary *in silico* testing. These may further elicit ideas and new hypotheses to be eventually tested *in vivo*. In several applications over recent years, the model has generated emergent, sometimes surprising, data that shed light on the mechanisms and interactions of the model itself and on their counterparts in the biological immune system. For example, during the simulation of the affinity maturation of the humoral response, the varying density of cells and availability of antigen were shown to cause the shift from the bottleneck of the primary response, obtaining the help of CD4^+^ cells, to the secondary bottleneck, winning the competition for antigen (11).

The model offers the possibility to manipulate the elements of virtual runs like experimental biologists do, by using the computational equivalent of knock-out mice or cell transfer (4, 12). Stratagems of this kind were applied in parallel experiments comparing the response of the humoral branch only, the cellular branch only, and both branches, to relate the efficiency of responses to different viral features (13). In a study about cross-reactive memory, the silencing of one or the other of two suspected kinds of attrition, active or passive, revealed interesting cooperative effects of the combined mechanisms (1). In another study, selective “freezing” of humoral cross-reactive responses was obtained by increasing the bit distance in epitopes but not in peptides, while the antibody lifetime was artificially shortened or extended over a 50-fold range in order to reveal antibody-mediated competition against cellular responses (3).

## Materials and Methods

### The Computational Model

#### Polyclonality

In the present computational model, the specific recognition in adaptive immunity is simulated by borrowing ideas from binary calculus (14). Epitopes and paratopes are represented by strings of zeros and ones. When an epitope meets a paratope the strings are checked for complementarity at each position and a match (or equivalently a mismatch) is scored. Thus, the match is a number between 0 and *N* where *N* is the length of the binary strings representing the two binding regions. The model is polyclonal since it equips cells and molecules (e.g., lymphocytes receptors, B-cell receptors, T-cell receptors, Major Histocompatibility Complexes (MHC), antigen peptides and epitopes, immuno-complexes, etc.) with specific bit strings to represent the “binding site.”

This minimalistic definition allows a diversity of 2^*N*^ for each immunocyte (CD4+ or Th, CD8+ or TC, B). Such a setup can model cross-reactivity with remarkable smoothness, and accuracy in predicting the effect of competition among cross reactive cells.

#### Binding Affinity

*In vivo*, the paratope-epitope attraction is the sum of weak electrostatic and hydrophobic interactions when juxtaposed. In the simulation, two entities interact with a probability that is a function of the Hamming distance between the binary strings representing the entities' binding site. We indicate with *m* = ||*r, p*|| ∈ {0 … *N*} the distance or the match between *r, p* ∈ {0 … 2^*N*^−1}. A good and widely used analogy is the matching between a lock and its key. If more than a threshold value *m*_*c*_ over *N* bits matches (i.e., 0–1 or 1–0) occur, the interaction is allowed with a certain probability that is a function of the number of matches between the bit-strings. This attraction force (called *affinity* or *affinity potential*) is equal to one when all corresponding bits are complementary. Specifically, if *m* = ||*r, p*|| is the Hamming distance between the two strings *r* and *p*, the affinity potential *f* (*m*) ∈ [0, 1] defined in the range 0, …, *N* is

(1) $$f\left(m\right)=f\left(\left\|r,p\right\|\right)=\{\begin{array}{c} e^{\text{log}(A_{L})\frac{m-N}{m_{c}-N}}\;m_{c}\leq m\leq N\\ \;0\;m<m_{c} \end{array}$$

where *A*_*L*_ is a free parameter which determines the slope of the function, whereas *m*_*c*_ ∈ {*N*/2 … *N*} is the cut-off (or threshold) value below which no binding is allowed.

#### Humoral and Cellular Responses

The model simulates a very simple form of innate immunity and an elaborate form of adaptive immunity (including both humoral and cytotoxic immune responses).

In the case of innate immune response by “exogenous signal” (e.g., Pathogen-Associated Molecular Pattern, PAMP or PAMP-agonist, used for specific adjuvants) the activation sequence will begin with antigen presenting cells stimulation. The only mechanisms of this kind which is embedded in the model accounts for the presence of lipopolysaccharides in pathogens as in Gram-negative bacteria.

#### Working Assumptions

In the model, a single human lymph node (or a portion of it) is mapped onto a three-dimensional Cartesian lattice. The primary lymphoid organs thymus and bone marrow are modeled apart: the thymus (15, 16) is implicitly represented by the positive and negative selection of immature thymocytes before they enter the lymphatic system, while the bone marrow generates already mature B lymphocytes. Hence, only immunocompetent lymphocytes are modeled on the lattice.

The C-IMMSIM model incorporates several working assumptions or theories, most of which are regarded as established immunological mechanisms, including: (i) the clonal selection theory of Burnet (17); (ii) the clonal deletion theory (i.e., thymus education of T lymphocytes) (18); (iii) the hypermutation of antibodies (19); (iv) the replicative senescence of T-cells, or the Hayflick limit (i.e., a limit on the number of cell divisions) (20); (v) T-cell anergy (21) and Ag-dose induced tolerance in B-cells (22); (vi) the danger theory (23); (vii) the idiotypic network theory (24). Variations on the basic model have been used to simulate different phenomena ranging from viral infection [e.g., Human Immunodeficiency Virus (25) or Epstein-Barr Virus (26)] to cancer immunoprevention and type I hypersensitivity (27, 28).

Each time step of the simulation corresponds to 8 h. The interactions among the cells determine their functional behavior. Interactions are coded as probabilistic rules defining the transition of each cell entity from one state to another. Each interaction requires cell entities to be in a specific state choosing from a set of possible states (e.g., naïve, active, resting, duplicating) that is dependent on the cell type. Once this condition is fulfilled, the interaction probability is the effective level of binding between ligand and receptor.

Unlike many other immunological models, the present one not only simulates the cellular level of the inter-cellular interactions but also the intra-cellular processes of antigen uptake and presentation. Both the cytosolic and endocytic pathways are modeled. In the model, endogenous antigen is fragmented and combined with MHC class I molecules for presentation on the cell surface to CTLs' receptors, whereas the exogenous antigen is degraded into smaller parts (i.e., peptides), which are then bound to MHC class II molecules for presentation to the T helpers' receptors.

#### Stochasticity

The stochastic execution of the algorithmic rules, as in a Monte Carlo method, produces a logical causal/effect sequence of events culminating in the immune response and development of immunological memory. The starting point of this series of events is the injection of antigen (the priming). This may take place any time after the simulation starts. In general, the system is designed to maintain a steady state of the global population of cells if no infection is applied (homeostasis). Initially the system is “naïve” in the sense that there are neither T and B memory cells nor plasma cells and antibodies. The various steps of the simulated immune response depend on what is injected, i.e., virus or bacteria.

#### The Virus

Virus is the “foreign agent” in the model. It is constructed with B-cell epitopes and T-cell peptides. In addition, it replicates, simulating a living entity, and the combination of three factors (speed of duplication, infectivity, and lethal load level) results in its “fitness” which is independent of antigenicity. Any infection begins with the penetration of virus into an epithelial cell, though this could be any designated target cell. Whether the infection is cured or becomes persistent or even kills the virtual mouse depends on the virus dose, its fitness, and the strength of the immune response it has elicited. All these variables determine whether—and to what degree—the immune system's success requires the cooperation of both the cellular and humoral branch, as has been shown in several simulation studies (13).

#### Modeling Active Attrition

Active attrition is enacted in the present version of the model by describing the release of IFN-β by macrophages in the presence of high concentrations of danger signals, e.g., in infection sites. This lymphokine diffuses locally and then “causes” the death of cytotoxic memory T-cells by contact. The locally-limited bystander effect of this cytokine is set to be dependent on the cell's age but also on its affinity to the viral peptide. Specifically, the death of cytotoxic cells is modeled as a stochastic event whose probability is proportional to the cell's age and inversely proportional to the affinity between TCR and the peptide attached to class 1 HLA (1, 5) of infected cells, i.e.,

(2) $$\begin{array}{l} \mathrm{Pr}\left[die\right]=\frac{a^{n_{1}}}{a^{n_{1}}+k_{1}}\times \frac{i^{n_{2}}}{i^{n_{2}}+k_{2}}\times \left(1-f\right) \end{array}$$

where *a* is the age of the T-cell (in units of days), *f* the affinity of its TCR to the viral peptide as defined in Equation (1) and *i* is the local concentration of IFN-β (in pg/ml). In the experimental setup that we are going to describe in the following section, the parameters of Equation (2) have been chosen as follows: $k_{1}=10^{6}\times {\text{days}}^{-1}$ and $k_{2}=10^{9}\times {\left(\text{pg}/\text{ml}\right)}^{-1}\;were$ taken to obtain a probability of killing which was much stronger for memory compared to naïve cells; parameter *n*_1_ = 3 > *n*_2_ = 2 were chosen to make age the limiting factor in the killing. The last term in Equation (2), (1−*f*) ∈ [0, 1], stands for a protective factor for cells able to establish a stronger immunological synapse during peptide recognition on the membrane of infected cells and *f* therefore is the same function in Equation (1).

### Experimental Setup

The model represents both paratopes and epitopes by *N* = 16 bit binary strings. A successful paratope-epitope interaction is limited to a match *m* greater than or equal to the cut off *m*_*c*_ = 13 over the 16 allowed. This setup results in a diversity of 2^16^ for each lymphocyte and gives *N* − *m*_*c*_ = 4 matching classes thus allowing to model the immune recognition and predicting the effect of competition among cross-reactive cells with reasonable accuracy. *In vivo*, the diversity among epitopes and that among paratope are mind boggling (conservatively, 10^10^ to 10^12^). Simulating those numbers, though theoretically possible by enlarging the repertoires which is obtained by elongating the strings, is practically not viable for computational reasons.

#### The Antigenic Distance Experiments

In studying memory, it is important to quantify the degree of cross-reactivity between related antigens. While *in vivo* this appraisal is difficult to attain, the following modeling setup allows us to measure the effect of cross-reactivity on a secondary immune response quite effectively.

The series of simulations we perform mimic a prime/challenge experiment in a virtual mouse (or individual) where successive injections carry equal or different antigenic determinants (see Supplementary Figure 2). The priming infection is performed always with the same virus, but the challenge or secondary infection performed later is done with a different virus whose peptide is at a defined distance *d* from the priming one. We use *N*/2 = 8 bits to represent a virus peptide thus we have *d* = 0 … 8 levels of cross-reaction by suitably choosing the prime/challenge couple. Viral peptides are presented to T-cell receptors bound to the major histocompatibility complex molecule (MHC) and indeed in the model the match is an *N*-bit match. However, for simplicity, the contribution to the affinity given by the portion of the cell receptor binding the portion of the MHC molecule is set to a constant value so not to influence the overall match to the virus. In other words, the affinity between receptors and MHC-bearing virus peptides depends only on a *N*/2 = 8 bit match rather than an *N* bit match.

Let's call *V*^*I*^ the virus injected first (i.e., the primer at time *t*_*I*_), *V*^*II*^ the virus injected subsequently (the challenger at time *t*_*II*_) and *d* the “bit distance” between *V*^*I*^ and *V*^*II*^, that is, *d* = ||*V*^*I*^, *V*^*II*^||. The experiments realize the protocol consisting in a priming injection that is always performed with the same virus ${V^{I}=V}_{0}$ and a challenge injection consisting of a certain saturating dose of one of the nine viruses reported in Table 1 which also includes *V*_0_. Therefore ${V^{\text{II}}=V}_{k}$ for *k* = 0 … 8. Note that the set of chosen viral peptides is such that *d* = ||*V*_*i*_, *V*_*j*_|| = |*i* − *j*|, for all choices of *i, j* ∈ {0 … 8}. Following this description, it is convenient to name the experiments on the basis of the distance between priming with *V*_0_ and challenge with *V*_*k*_. For instance, we call *d* = 3 the experiment in which $V^{\text{I}}=V_{0}$ and $V^{\text{II}}=V_{3}$ because $d=\|V^{\text{I}},V^{\text{II}}\|=\|V_{0},V_{3}\|=3$. While *d* = 0 realizes the *homologous response*, and can indeed be considered the *control*, *d* = 1 to *d* = 6 represent cases of cross-reactivity, with progressively fewer matches. Finally, *d* = 7 and *d* = 8 are *heterologous* responses (i.e., no match at all). We note that all viral peptides are chosen to be distant with respect to self-peptides, to avoid having to deal with autoimmune responses, which are outside the scope of this work.

**Table 1:** Viruses used in the experiments are numbered from zero to eight.

**V_k_**	**Peptide strings(p_k_)**
*V*_0_	0000 0000
*V*_1_	0000 0001
*V*_2_	0000 0011
*V*_3_	0000 0111
*V*_4_	0000 1111
*V*_5_	0001 1111
*V*_6_	0011 1111
*V*_7_	0111 1111
*V*_8_	1111 1111

*The injection protocol comprises two viruses V^I^ and V^II^ presented one after the other at time steps separated by a time sufficient to fully develop an immune response. The Hamming distance between the two viruses injected determines the level of cross-reactivity hence the degree of exploitation of the immune memory to V^I^ in the response to V^II^. Viruses to are equipped with a peptide string of length N such that d = ||V^I^, V^II^|| ϵ{0 … 8}. In the antigenic distance experimental protocol V^I^ is always equal to V_0_*.

The simulated space is equivalent to a fraction of the lymphatic system represented at once. This simulated volume is 10 micro liters or, equivalently, 10 cubic millimeters. Both priming and challenge consist in injecting a *saturating* viral dosage of 10^3^ viral particles per microliter. For all experiments, the setup is identical except for the two viruses injected, *V*^*I*^ and *V*^*II*^. Thus, the simulated space is populated with the same initial number of cells (i.e., no variability allowed), the viruses share the same infectivity and replication rates, etc. Moreover, since the model is stochastic, for each setup *d* ∈ {0 … 8}, we repeat the experiments 100 times for each protocol and calculate statistics (averages and standard deviations) afterwards.

#### Useful Definitions

With the aim of defining two quantities which help in measuring the effect of cross-reactivity, we now need to introduce some formalism.

We call *diversity D* the set of possible bit strings of length *N* in the base-ten system, that is, *D* = {0…2^*N*/2^ − 1}. We indicate by *n*_*r*_(*t*) the number of cytotoxic T-cells with specificity *r* ∈ *D* at time *t*. For each virus *V* the Hamming distance creates the equivalent classes in the set of cell receptors *D*. In other words, two receptors *r*_1_ and *r*_2_ are in the same matching class for *V* if ||*r*_1_, *V*|| = ||*r*_2_, *V*|| = *m*. We can therefore define *q*_*m*_(t) as the total number of cells matching the virus *V* with *m* bits, that is, ∀*m* ∈ {0 … *N*}

$$\begin{array}{l} q_{m}\left(t\right)=\underset{r\in D,\|r,V\|=m\;}{\sum }n_{r}\left(t\right). \end{array}$$

Then we call *A*_*m*_(*t*) the affinity of the response to the peptide of virus *V* relative to the matching class *m* ∈ {0 … *N*}, that is, all the lymphocytes that are equivalent in terms of affinity. This quantity is calculated by summing the number of cells with receptor matching with *m* bits the virus peptide and multiplied by the affinity value *f*(*m*), that is, ∀*m* ∈ {0 … *N*}

(3) $$\begin{array}{l} A_{m}\left(t\right)=f\left(m\right)\cdot q_{m}\left(t\right). \end{array}$$

Finally, we define the total affinity to virus *V* as

(4) $$\begin{array}{l} TA\left(t\right)=\overset{N}{\underset{m=0}{\sum }}A_{m}\left(t\right). \end{array}$$

Note that since we are interested in quantifying the effects of cross-reactivity on the secondary immune response, all the quantities *q*_*m*_(*t*), *A*_*m*_(*t*) and *TA*(*t*) should be considered relative to *V*^II^ and be written, for instance, $A_{m}^{II}$. However, to simplify the representation we just avoid using the superscripts and write *A*_*m*_, etc.

Furthermore, we call *V*^*II*^ (*t*) the number of viral particles at time *t* of the challenge virus and define

$$\begin{array}{l} t_{c}=\underset{t}{\min}\left\{t\geq t_{II}:V^{II}\left(t\right)=0\right\}-t_{\text{II}} \end{array}$$

the time-to-clear the virus *V*^*II*^, that is, a measure of how quickly the response eradicates the virus injected at time *t*^II^.

We can now finally define two almost complementary measures. The first one is the *efficacy* of the immune response to the second virus injected *V*^II^. The efficacy *E*(*d*) is a function of the distance

$$\begin{array}{l} d=\|V_{0},V^{\text{II}}\| \end{array}$$

to the first injected virus $V^{I}=V_{0}$ and is defined as the *peak value* of *TA*(*t*) for *t* ≥ *t*_*II*_ divided by the time-to-clear the virus *t*_*c*_. In formula

(5) $$\begin{array}{l} E\left(d\right)=\frac{1}{t_{c}}\cdot \underset{t\geq t_{\text{II}}}{\max}\left\{TA\left(t\right)\right\}. \end{array}$$

The efficacy measures how good the immune response to *V*^*II*^ is in terms of how many cytotoxic T-cells are developed by clone expansion and how quickly the virus is eliminated. Clearly the maximum value of the efficacy is achieved for *d* = 0 because of the immune memory developed to respond to $V^{I}=V_{0}$, but decreases for increased distance *d* between prime *V*_0_ and challenge injection *V*^*II*^.

The maximum value attained by the sum of all Tc counts *q*_*m*_(*t*) for *m* = 0 … *N* averaged over a number of simulations (〈·〉 indicates averages) can be designated as

(6) $$\langle \tilde{M}\rangle =\langle \underset{t\geq t_{\text{II}}}{\max}\left\{\sum_{m=0}^{N}qm(t)\right\}\rangle .$$

Cell counts are calculated for each antigenic distance experiments. We can therefore use superscripts to indicate a specific experiment and refer to this quantity in the case *d* = 8 as ${\langle \tilde{M}\rangle }^{d=8}$. This value measures the magnitude of the cytotoxic immune response to $V^{II}=V_{8}$. Since it corresponds to the completely heterologous response, the effect of the MaN is zero and the quantity in Equation (6) is *maximal* with respect to *d*. The other extreme case is found when *d* = 0, corresponding to a homologous immune response for which the immune memory is so perfectly fit to the second injected virus $V^{II}=V_{0}=V^{I}$ that the latter is eliminated without the need for a clonal expansion of cytotoxic T-cells. The measure that we call *compression* is then defined as

(7) $$\begin{array}{l} C\left(d\right)={\langle \tilde{M}\rangle }^{d=8}-{\langle \tilde{M}\rangle }^{d} \end{array}$$

and is the difference of the maximum number of cytotoxic T-cell count attainable in the absence of memory. In other words, this measure quantifies the degree of hindrance (or reduction, hence the name compression) of the naïve response due to the presence of cross-reactive memory cells against past infections. The compression is maximal for *d* = 0 and diminishes for larger *d* reaching its minimum for *d* = 8.

## Results

### The Memory Anti-naïve (MaN) Phenomenon

**Figure 1:**
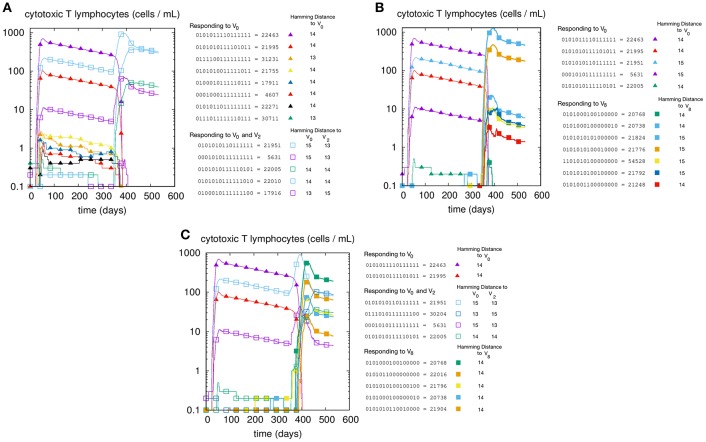
T-cell counts corresponding to three different but similar simulations. In panel **(A)** we inject two homologous viruses (i.e., cross-reacting) in succession (i.e., at day *t*_*I*_ = 0 and *t*_*II*_ = 330) namely ${V^{I}=V}_{0}$, $V^{II}=V_{2}$, which are therefore at a distance of 2 bits. In panel **(B)** we inject two heterologous viruses, *V*_0_ and *V*_8_. In panel **(C)** instead, after the usual priming with *V*_0_ we challenge the system with both the homologous *V*_2_ and the heterologous *V*_8_ viruses. Cell counts shown in all panels are representative of the most active clones of the 2^*N*^ possible. These “responding clones” are specific either to just *V*_0_ (filled triangles in all panels **A–C**), or specific to both *V*_0_ and *V*_2_ (i.e., cross-reacting memory clones shown in panels **A,C** as empty squares), or naïve independent responses specific to *V*_8_ (panels **B,C**, filled squares). In panel **A** the primary naïve response to V_0_ (filled triangles) leaves memory that is then re-stimulated by *V*_2_. In panel **B** instead, the same primary naïve response to *V*_0_ (filled triangles) do not cross-react to *V*_8_ and indeed fade away upon the raising of the secondary responses. **(C)** shows what happen when the system is challenged with both. It shows in particular that the heterologous response to *V*_8_ (filled squares) is independent and unaffected by the cross-reacting clones (empty squares) operating the MaN.

We first illustrate the MaN phenomenon by studying the primary and memory responses against viruses with different antigenic distance. The results of three cross-reacting viral infections are shown in panels A, B, and C of Figure 1, where we can track the primary and memory responses of proliferating individual T-effector memory clones. In each panel we learn the composition of the naïve response, represented by filled markers, and of the memory response, represented by empty markers present only in panels A and C. Panel A shows the case of priming with $V^{I}=V_{0}$ and challenge $V^{II}=V_{2}$, that is, a virus with antigenic distance *d* = 2 that elicits a cross-reactive memory response. The result is a strong dominance of memory over primary clones. In fact, no new primary clone emerges after *t*_*II*_. Panel B has a priming identical to that of panel A but is challenged by a virus with *d* = 8 from the priming, thus a heterologous virus. As predicted, the secondary response does not trigger memory cells, but elicits a naïve response specific to the challenge *V*_8_. Panel C shows the case with all three viral infections: *V*_0_ at time *t*_*I*_, producing a primary stimulation, and both *V*_2_ and *V*_8_ at time *t*_*II*_. The two latter viruses are, by virtue of their antigenic distance, not interfering with each other. Any primary anti-*V*_2_ is silenced by cross reacting memory previously elicited against *V*_0_, while the naïve anti-*V*_8_ mounts, as expected, an undisturbed primary response. Taken together, these results allow us to conclude that the force underpinning MaN is specificity, and the mechanism is competition for antigen. Note that both panels A and C show a clear advantage of memory over naïve: most memory clones are higher than naïve ones at the peak.

### MaN Has Two Different Effects

Another way of showing MaN is to extend the range of distances between the priming and the secondary infection, that is, following the antigenic distance experiment schema described in section The Antigenic Distance Experiments. In this set of experiments the attrition plays no role as it has been disabled, allowing us to study the MaN in isolation.

**Figure 2:**
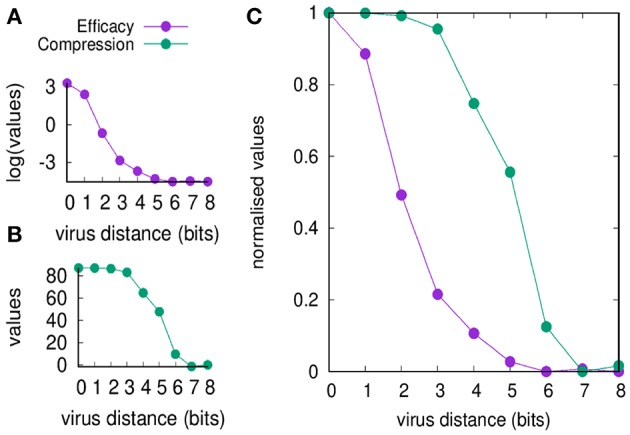
Memory response clears the virus and dominates over any primary response by its faster action. This figure shows that the domination remains in place also when the antigenic distance *d* makes any cross-reactive memory response weak. In panel **(A)**, we show the efficacy of the immune response *E*(*d*) (i.e., a measure of cytotoxicity in unit of cell counts) vs. the antigenic distance *d*. In panel **(B)**, the compression *C*(*d*) (i.e., the blocking effect of the cross-reactive memory, also in unit of cell counts). The difference of the curves indicates that for *d* ∈ {2 … 5} the blocking of the primary response is not justified by an efficient secondary response, that is the phenomenon that constitutes the MaN. Panel **(C)** presents the same data of panels **(A,B)** but respectively normalized by the Min-Max method (i.e., $y^{\prime }=\left(y-y_{\min}\right)/\left(y_{\max}-y_{\min}\right)$) so to fall within the same range [0, 1]. It visualizes at once the effect of MaN as the area between the two curves assuming the shape of an “eye”.

Figure 2 shows the efficacy *E*(*d*) and the compression *C*(*d*) defined in section Useful Definitions [respectively in Equations (5) and (7)] as a function of the viral distance *d*. The figure shows values before and after normalization. Clearly, the efficacy of the immune response decreases by increasing *d* (panel A) simply because it is a measure of the efficiency of the cross-reactive immune memory. We can point to *d* = 2 as the critical value for the greatest reduction in efficacy. On the other hand, the compression also decreases by increasing *d* for the same reason, but changes more “slowly” compared to the efficacy: the critical value is *d* = 5. Based on these results, we can say that the range *d* between 2 and 5 is the *domain* of the MaN.

### The Effect of Attrition in Alleviating the MaN

In another set of experiments, we turned the attrition on and studied its effect on the immune response in general and on the MaN in particular. The attrition effect was modulated by modifying Equation (2) by multiplying the interferon concentration by a factor α, that is, taking $\alpha \times i^{n_{2}}$, with α equal to 1,2, … 5. For formal correctness, the case of no attrition designated as α = 0 needs to be made explicit in the new definition of Pr[*die*] of Equation (2) as follows

(8) $$\begin{array}{l} \mathrm{Pr}\left[die\right]=P\left(\alpha \right)=\frac{a^{n_{1}}}{a^{n_{1}}+k_{1}}\times \frac{\alpha i^{n_{2}}}{\alpha i^{n_{2}}+k_{2}}\times \left(1-f\right) \end{array}$$

**Figure 3:**
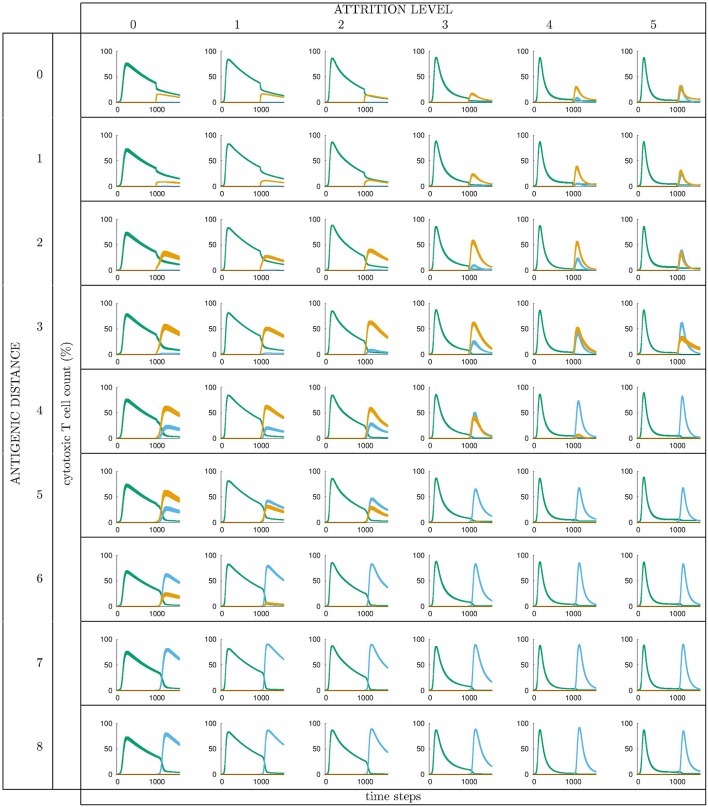
Cytotoxic cell counts (percentage) vs. time. This is the result of different simulations obtained varying the level of attrition α = 0 … 5 (α = 0 is control case of no attrition) and the antigenic distance *d* between the two viral infections, for a total of 6 ×9 = 54 panels, each containing average ± standard deviation results of Tc counts in simulated primary viral infections, followed by a second challenge infection by an identical, or by a selected mutant virus. Color codes: *green*: response by naïve T effector cells to primary virus *V*^*I*^ injected at *t*_*I*_ = 0; *orange*: cross-reactive memory response primed by the first virus *V*^*I*^ and challenged by *V*^*II*^; *blue*: response by naïve T effector cells to *V*^*II*^ injected at *t*_*II*_ = 1000 time steps. See text for further explanation.

**Figure 4:**
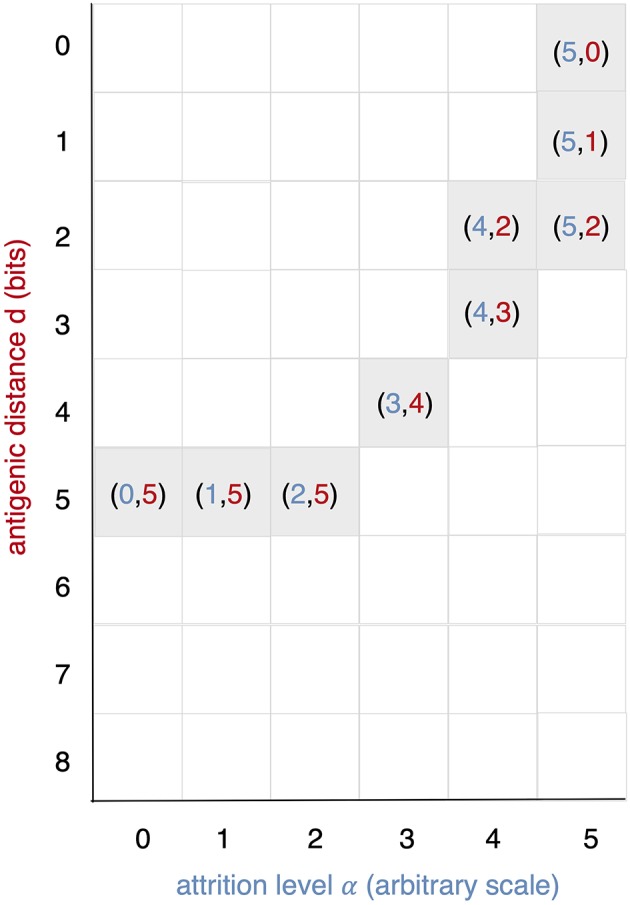
This diagram is meant as a visual help to the analysis of Figure 3 and keeps on the same coordinates: virus distance (*d* = 0 … 8) vs. attrition level (α = 0 … 5). In Figure 3 we see that memory is strong and dominant but can be trimmed down by turning two independent knobs: the first increases *d* and affects affinity, the second through *attrition*, thins the memory cell population, by inducing apoptosis. If both knobs are turned up in the same run a synergic effect is observed. The equal counts of memory and naive cells are due to the fact that in the clearing of the virus that in the clearing of the virus the total affinity required is contributed equally by the two populations.

All runs exhibit identical primary response to *d* = 0 virus (green). Analyzing the memory responses those challenged with *V*_0_ (i.e., *d* = 0 thus homologous) are the strongest while all others are cross-reactive and expected to turn out progressively weaker with increasing *d*. For *d* = 7 and *d* = 8 the viral challenging epitope is so different from the priming epitope that memory fails to recognize it, thus there is no cross-reactive memory and the response is a primary response directed against the second virus (*V*_7_ or *V*_8_). Another point that may seems counterintuitive is to see a very weak immune response representing the fact that when the memory is at its strongest (e.g., *d* = 0 and *d* = 1) the virus is eliminated very efficiently. This happens because memory is “speedy in deployment” and eliminates the growing population of pathogens when they are still few in numbers. This has two important effects: (i) the lack of further stimulation keeps the effectors low, and (ii) no stimulation of naïve response takes place. This is the same competition for antigen already seen in Cheng et al. (3). The results in Figure 3 quantitatively confirm these early observations and provide further insights into the mechanisms. Either large antigenic distances or high levels of attrition will counter the MaN effect but, as expected for synergistic actions, smaller antigenic distances or levels of attrition result in a *balance* between memory and naïve total affinity. This is visible in several cases of the grid in Figure 3. To simplify the study of these “ties,” and the eventual takeover by naïve responses, the position of the critical runs of Figure 3 are pinpointed in Figure 4. In eight cases ties between primary and memory occur, at the point where the primary curve is about to surpass the memory response. Since all primary responses are identical against any virus, in these eight cases we know that the *total affinity* [i.e., *TA*(*t*) of Equation (4)] of memory is comparable to the total affinity of the primary response. Surpasses are easy to spot in Figure 3, following the coordinate marked on Figure 4, where distance and attrition are color coded in red and blue, respectively.

Results for B cells approximately follow those shown for T cells (see Supplementary Figure 1). This was expected since in the definition of viruses *V*_0_ … *V*_8_ we have purposefully followed the same logic (i.e., distance) in the definition of the viral epitopes with the aim of not favoring the humoral response of one virus in particular. The conclusion is that, with regards to the balance between MaN and attrition, the humoral response is consistent (i.e., is very similar) to the cytotoxic response and, in substance, it does not prevent or limit the latter but adds to it instead.

**Figure 5:**
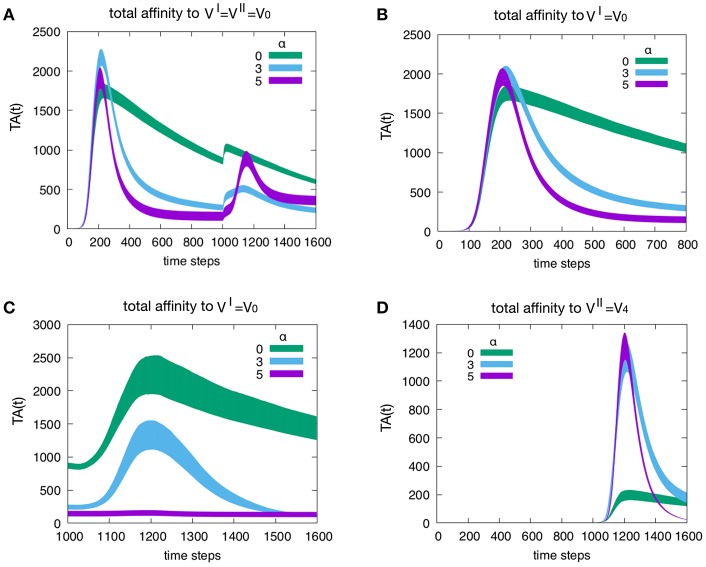
Effect of attrition on the Tc total affinity *TA*(*t*). Panel **(A)** shows how the attrition influences the total affinity in a *d* = 0 experiment, i.e., the homologous response to $V^{I}=V^{II}{=V}_{0}$. Only three levels of attrition shown. The highest level of attrition (α = 5) does not affect the peak of memory (in the homologous response the second peak is due to memory recall) but trims the curve via its effect on aged cells. In other words, as expected, the attrition facilitates the emergence of higher affinity cells thus increasing the peak values of the total affinity especially in the primary response. This will be shown more clearly in Figure 6. Panel **(B)** When simulating a heterologous response (i.e., heterologous challenge $V^{II}=V_{4}$ thus for viral distance *d* = 4), the total affinity to the V^I^ = *V*_0_ is equivalent to the one in panel **(A)**. Panel **(C)** shows the same total affinity to $V^{I}=V_{0}$ but calculated at the time of the challenge that is during the competing presence of anti-*V*_4_ naïve cells. Here, by increasing the level of attrition, the memory to $V^{I}=V_{0}$ disappears; this effect is striking when compared to the absence of attrition case of the green curve. Panel **(D)** once more shows the total affinity in a *d* = 4 experiment but this time relative to the virus injected as challenge, i.e., $V^{II}=V_{4}$. The comparison of the total affinity should be made with respect to the peak values for *t*>1000 in panel A. Clearly, attrition has favored the emergence of higher affinity clones (blue and purple corresponding to α = 3 and 5, respectively) with respect to the absence of affinity case (green curve).

**Figure 6:**
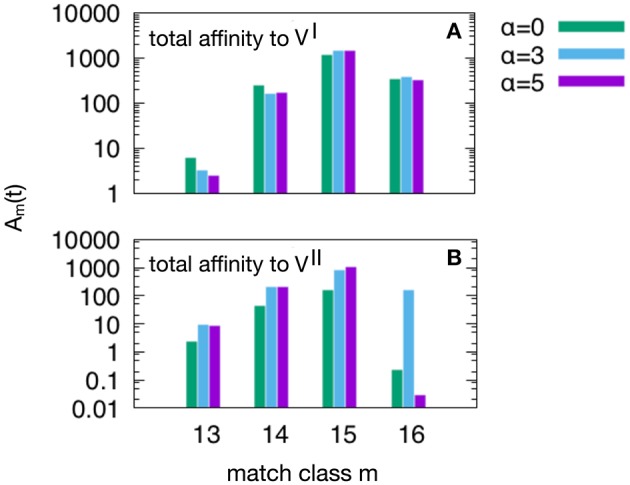
This plot show the peak values of *the A*_*m*_(*t*) per match classes, from the lowest *m* = *m*_*c*_ = 13 to the highest *m* = *N* = 16. As in Figure 5, we have three attrition levels α = 0 (corresponding to absence of attrition), α = 3 (considered the “optimal” level) and α = 5 (deemed a excessive value for attrition) and we run the *d* = 4 experiment meaning that $V^{I}=V_{0}$ and $V^{II}=V_{4}$, because this case is the one in which the MaN and the attrition display the greatest effects. The first observation is that *m* = 15 is the match class which reaches the highest peak values. This is expected given the relatively short time to develop a complete affinity maturation during either the primary response (panel **A**) and the secondary heterologous response (panel **B**). Another observation pertaining the lower panel is that the attrition helps improving the affinity maturation in all match classes. Moreover, while attrition α = 3 helps the maturation of *m* = 16 high affinity clones, the case α = 5 knocks them down (panel **B**).

Figure 5 shows the effect of attrition on the Tc total affinity *TA*(*t*) defined in Equation (4). Panel A refers to how attrition influences the total affinity of a homologous response, i.e., when to $V^{I}=V^{II}=V_{0}$ resulting in *d* = 0. Only three levels of attrition are shown: α = 0 corresponding to absence of attrition, α = 3 considered the intermediate “optimal” level and α = 5 deemed an excessive value for attrition. The highest α = 5 does not affect the peak of memory since in the homologous response the second peak is due to memory recall but trims the curve via its effect on aged cells. As expected, attrition facilitates the emergence of higher affinity cells thus increasing *TA*(*t*) especially in the primary response. This is better shown in Figure 6. Panel B unveils the heterologous response (i.e., $V^{II}{=V}_{0}$ thus *d* = 4), the total affinity to the $V^{I}=V_{0}$ is equivalent to the one in panel A. Panel C shows the same total affinity to $V^{I}=V_{0}$ but calculated at the time of the challenge, namely, during the competing presence of anti-*V*_4_ naïve cells. Here, by increasing α, the memory to $V^{I}=V_{0}$ disappears. This effect is striking when compared to the case α = 0 (green curve). Panel D shows the total affinity in a *d* = 4 experiment but this time relative to $V^{II}=V_{4}$. The comparison of *TA*(*t*) should be made for *t* > 1000 in panel A. The overall message is that attrition favors the emergence of higher affinity clones (blue and purple curves in panel D corresponding to α = 3 and 5 respectively) with respect to the green curve (α = 0).

Figure 6 shows the peak values of *A*_*m*_(*t*) as defined in Equation (3) per match class, from the lowest value of match *m*_*c*_ = 13 to the highest *N* = 16. As in Figure 5 we have three attrition levels, α = 0, 3, 5 and we run the *d* = 4 experiment meaning that $V^{I}=V_{0}$ and $V^{II}=V_{4}$, because this case is the one in which the MaN and the attrition display the greatest effects. The first observation is that the class *m* = 15, although not the best match, reaches the highest affinity peak value. This is expected given the relatively short time to develop a complete affinity maturation during either the primary response (panel A) and the secondary heterologous response (panel B). Another observation pertaining to the lower panel is that the attrition helps improving the affinity maturation in all match classes. Moreover, clearly visible in panel B, while attrition α = 3 helps the maturation of *m* = 16 thus the high matching clones, the case α = 5 knocks them down. This comparison of levels of attrition suggests that the α = 3 has been correctly dubbed the moderate or optimal level.

## Discussion and Conclusions

Viral infections and pandemics are prime examples of the dynamic between evolution and mutability of viruses on one side and cross-reactivity of antibodies and cytotoxic cells on the other. Pandemics are often the result of recurrent infections with distant cross-reactive agents. The “original antigenic sin” (2) hypothesis, that is, the case of patients whose memory responds to a previous priming and whose primary response is blocked by memory, still lacks an explanation for why cross-reactive anti-virus cells that unable to clear the virus, are still able to outcompete the naïve cells. More recently, Monsalvo et al. (29) found signs of antigenic sin in non-protecting antibodies and low affinity immune complexes.

In this study we obtained quantitative data that enables us to propose a plausible mechanistic explanation for this phenomenon. We measured the ability of the immune system to deal concurrently with two viruses, one cross-reactive that is eliminated by memory cells that, as a side effect, block the naïve response, and another that is not cross-reactive and is eliminated by a naïve independent response (Figure 1). This shows that the two processes do not interfere with each other.

It is therefore the degree of cross-reactivity that determines the engagements of different concurrent immune responses. When the antigenic distance between the priming and the challenging virus is increased, memory efficacy *E*(*d*) falls immediately while the compression of naïve response *C*(*d*) through antigen deprivation is affected only later (see Figure 2). This result is expressed in the combination of the two curves of *C*(*d*) and *E*(*d*) as a function of antigenic distance, producing the iconic image of an “eye,” a representation and measure of MaN.

The efficacy *E*(*d*) is sensitive to decreased cross-reactive affinity, immediately from the first step and continues the descent as a concave curve, as expected in a cellular response where each cell's affinity contributes individually to the final result.

The compression *C*(*d*) shows no effect whatsoever in the first three steps decrease of affinity, while the fourth step causes only partial block of the naïve engagement. This resistance to severe decrease of affinity gives a measure of the dominance exerted by memory over naïve responses by depriving them of the antigen required for their growth. The display of strength by memory is certainly sustained by its speed of deployment, and this experiment detects a second concurrent mechanism that materializes as a cooperative action. Affinity is the energy displayed by a single paratope, but in a competition for the antigen, the presence of many paratopes nearby may decrease the chances of the antigen to “escape” from another memory cell. The best example is the higher “catching” ability of bivalent and pentavalent antibodies compared to Fab monovalent antibodies. To mark this difference the serologists of the last century invented a new strength inclusive of affinity and a “cooperation bonus” and called it *avidity*. Mechanistically two Fabs will bind two monovalent epitopes, but the weak forces will alternate periods of sticking together with period of detachment. Chances are that the two couplets will not stay in this conformation for long. On the other hand, the bivalent Fab has a definitely higher chance of staying with one epitope bound or at least, at short distance, quite stably. Avidity enhances binding and allows low quality memory cells to still dominate.

Any immune response, and particularly the fast, cellular memory is always in need of space (e.g., physical space, metabolic space, etc.), which like other resources are subject to competition. Active attrition (5, 30) consists of timely secretion of IFN-β operated by the same vectors that signal danger, and has the effect of eliminating crowding of cells by allowing a selection of more efficient young clones, at the expense of dominant clones. In this case, the action of the attrition has the specific connotation of helping the specific response as the thinning of clonal population will favor clonal expansion randomly. Based on the results shown in Figure 2, we propose that the difference in strength between antigen biding and compressing naïve cells depends on the advantage in favor of the latter: avidity is affinity enhanced by intra-clone paratope synergisms.

We have shown that the memory anti-naïve effect, the “necessary” byproduct of memory, can be mitigated by the attrition signals produced during the early stages of an infection. These signals kill a fraction of the population of effectors (Figures 5, 6). While resulting in a decreased speed of the response, this mechanism gets rid of low affinity cross-reactive cells thus allowing naïve clones to emerge and eventually achieve better affinity maturation.

In conclusion, the trimming effect of attrition which mitigates the MaN effect is well-documented in the present work and corroborates earlier studies (1, 4, 31). The present data is more precise as it independently monitors memory and naïve cells thus facilitating the detection of a phenomenon that affects different cellular compartments in opposite directions.

The results of this study add new predictions on the mechanism underpinning memory's clonal dominance on naïve responders: the competition is based on affinity to viral antigen, enhanced in the case of memory, by two factors, speed of action and intra clonal cooperation, resulting in the deprivation of antigen for naïve cells. We predict that clonal competitions are at the core of many pathologies that will not be understood and treated properly without explaining all causative forces.

In conclusion, results produced by computational models, however reasonable they may look, must be confirmed by *in vivo* or *in vitro* experiments before being considered scientific truth. However, their value may be realistically appraised if they trigger new hypotheses, and help guiding wet lab research. In this regard we believe that the presented modeling study has indeed provided a clearer picture of the complex relationship between MaN and attrition.

## Author Contributions

FrC contributed conception and design of the study. FiC performed numerical simulations and statistical analysis. DG and FrC contributed ideas during analysis and interpretation of the results. FrC wrote the first draft of the manuscript. DG and FiC wrote sections of the manuscript and revised the first draft. All authors contributed to manuscript revision, read and approved the submitted version.

### Conflict of Interest Statement

The authors declare that the research was conducted in the absence of any commercial or financial relationships that could be construed as a potential conflict of interest.
