# Supplementary material for: Computer Modeling of Clonal Dominance: Memory-Anti-Naïve and Its Curbing by Attrition
Source: Front Immunol. 2019 Jul 3;10:1513. doi: 10.3389/fimmu.2019.01513 (PMC6626922; doi:10.3389/fimmu.2019.01513)
Supplement: Supplementary file 1 [file Data_Sheet_1.PDF]

Supplementary Material

Computer Modeling of Clonal Dominance: Memory-Anti-Naïve and Its Curbing by Attrition

Filippo Castiglione[1], Dario Ghersi[2], Franco Celada[3*]

[1]Institute for Applied Computing, National Research Council of Italy, Rome, Italy

[2]School of Interdisciplinary Informatics, College of Information Science & Technology, University of Nebraska at Omaha, Omaha, NE, USA

[3]NYU School of Medicine, New York, NY, USA

***Correspondence:**
Franco Celada
Franco.Celada@nyumc.org

1 Supplementary Data

We have put in a github repository (`https://github.com/FilCast/cimmsim-exe`) the model (executable for Intel/Linux architectures) together with the input parameter files defining all protocols tested so that the interested reader can repeat the experiments.

The repository file contains the executable of the immune system simulator used in the article and compiled for Intel/Linux machines as follows:

```
cimmsim: ELF 64-bit LSB executable, x86-64, version 1 (SYSV), dynamically
linked, interpreter /lib64/ld-linux-x86-64.so.2, for GNU/Linux 2.6.32,
BuildID[sha1]=e3c73509535be14ba88692ffb14f2b9c86329938, not stripped
```

To execute it type the command:

```
cimmsim -f d?
```

where d? is one of d0, ..., d8

The results will be in the folder OUTDIR/ORGAN_0

For a description of the content of the files refer to related publications. ASCII space-separated files - details contain the most important cumulative information. One file for each agent-cellular entity. Each file has a header that describes its content.

A scheme of the viral-injection protocols used in the experimental setup of the article is provided in the Supplementary Figure 2.

1.1 Supplementary Figures

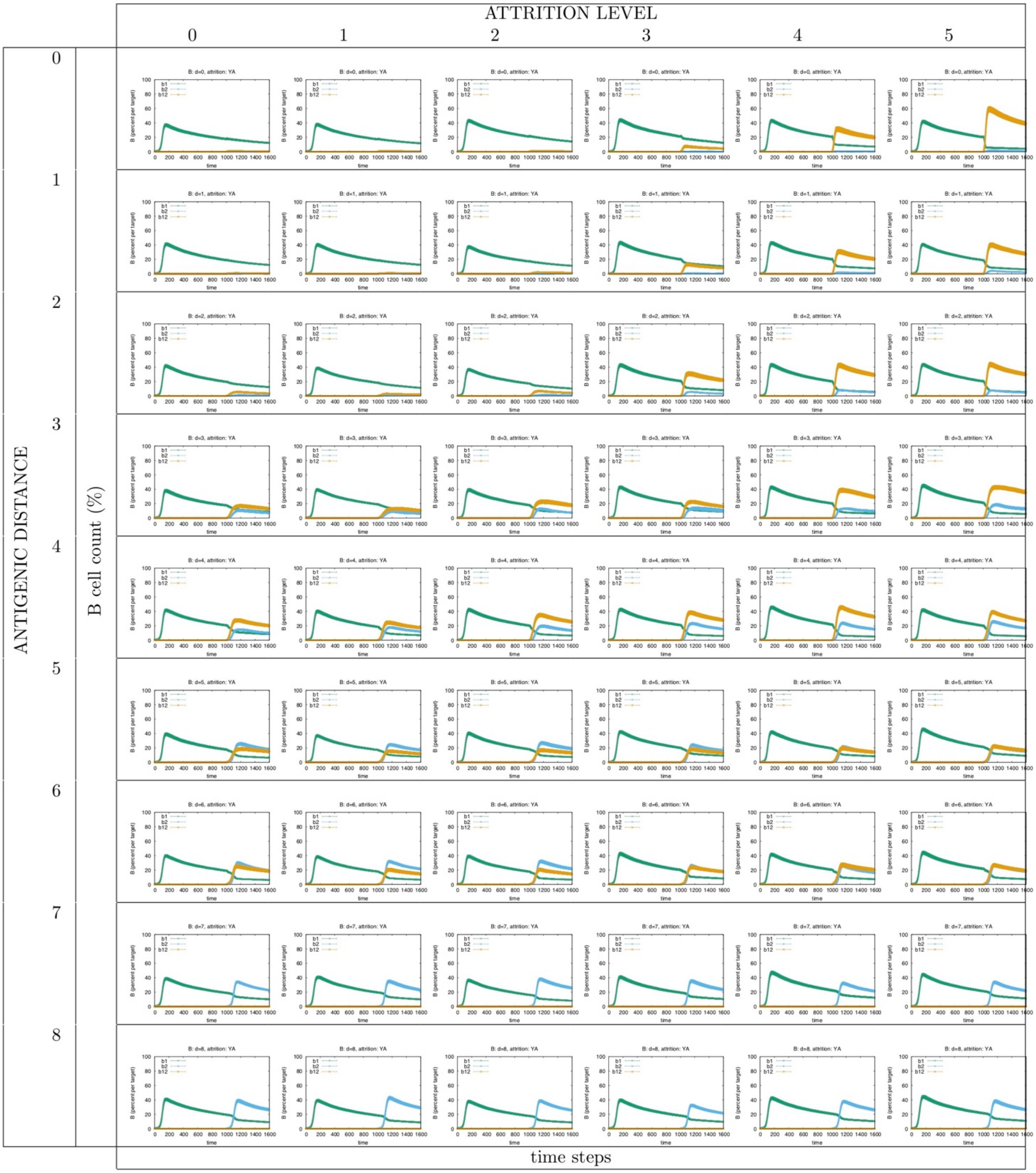

Supplementary Figure 1. Lymphocytes B counts (percentage) versus time. This is the result of the same set of simulation shown in figure 3 of the main text that have been obtained varying the level of attrition $\alpha = 0 \ldots 5$ ($\alpha = 0$ is control case of no attrition) and the antigenic distance d between the two viral infections, for a total of $6 \times 9 = 54$ panels, each containing average $\pm$ standard deviation results of B-cells counts in simulated primary viral infections, followed by a second challenge

infection by an identical, or by a selected mutant virus. Color codes: *green*: response by naïve B effector cells to primary virus V^I injected at $t_I = 0$; *orange*: cross-reactive memory response primed by the first virus V^I and challenged by V^{II}; *blue*: response by naïve B effector cells to V^{II} injected at $t_{II} = 1000$ time steps.

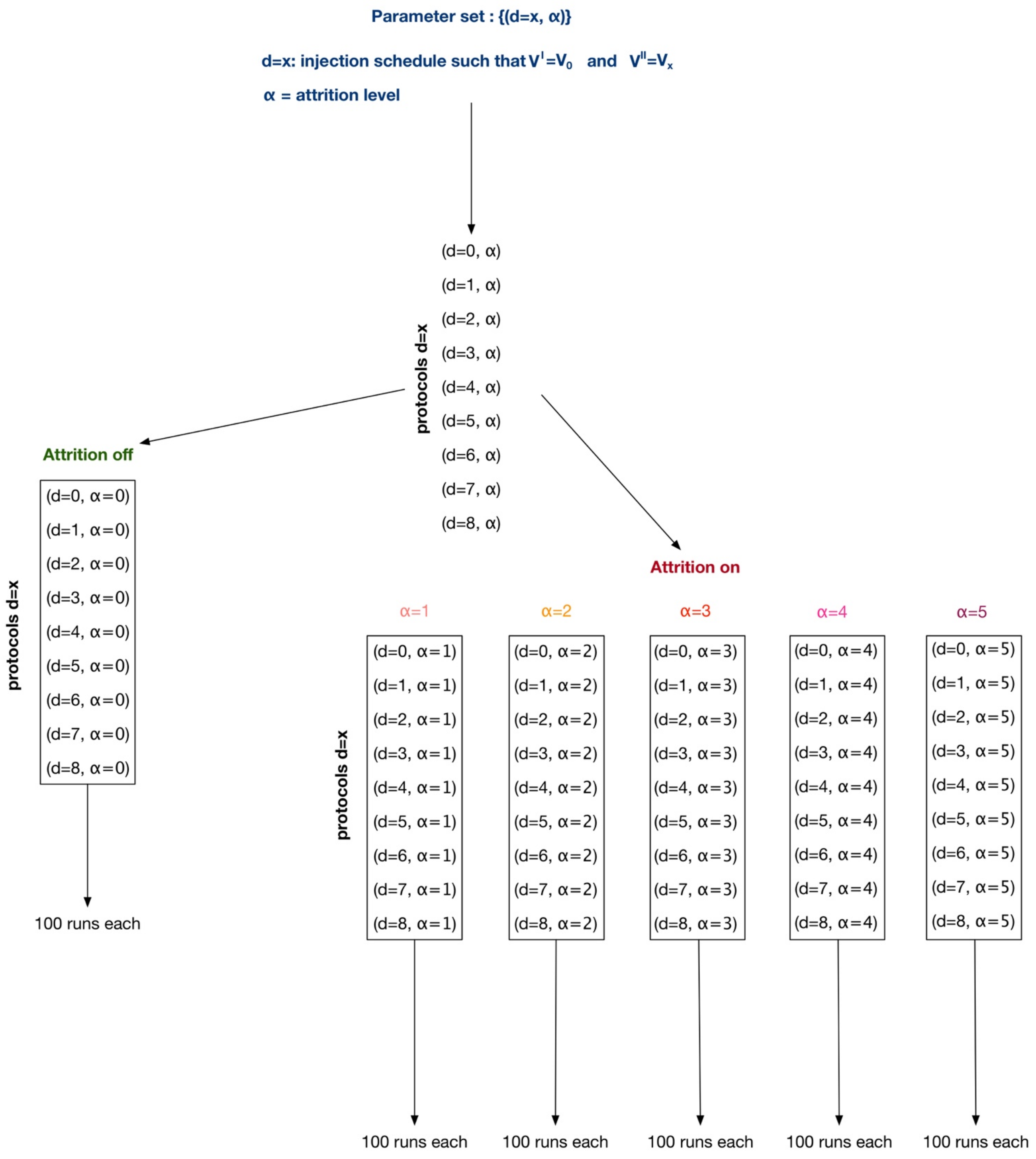

Supplementary Figure 2. Scheme of the viral-injection protocols used in the antigenic distance experiment described in section 3.2.1 of the main article.
